# Cultivable anaerobic and aerobic bacterial communities in the fermentation chambers of *Holotrichia parallela* (coleoptera: scarabaeidae) larvae

**DOI:** 10.1371/journal.pone.0190663

**Published:** 2018-01-05

**Authors:** Zhen-yu Zhang, Yimin Yuan, Muhammad Waqar Ali, Tao Peng, Wei Peng, Muhammad Fahim Raza, Yongshun Zhao, Hongyu Zhang

**Affiliations:** 1 College of Environmental Design, Wuhan Institute of Design and Sciences, Wuhan, Hubei, P. R. China; 2 State Key Laboratory of Agricultural Microbiology, Institute of Urban and Horticultural Pests, College of Plant Science and Technology, Huazhong Agricultural University, Wuhan, Hubei, P. R. China; MJP Rohilkhand University, INDIA

## Abstract

As important pests, scarab beetle larvae survive on plant biomass and the microbiota of the fermentation chamber play an important role in the digestion of lignocellulose-rich diets. However, the cultivable microbes, especially the anaerobic cultivable microbes, are still largely unknown. Here, both cultivable anaerobic and aerobic bacterial communities associated with the fermentation chamber of *Holotrichia parallela* larvae were investigated. In total bacteria cells directly enumerated by the 4’, 6-diamidino-2-phenylindole (DAPI) staining method, the viable plate counts of cultivable bacteria in the fermentation chamber accounted for 0.92% of proportion. These cultivable bacteria were prone to attach to the fermentation chamber wall (88.41%) compared to the chamber contents. Anaerobic bacteria were dominant in the cultivable bacteria attaching to the fermentation chamber wall (70.20%), while the quantities of anaerobes and aerobes were similar in the chamber contents. Polymerase chain reaction-denaturing gradient gel electrophoresis (PCR-DGGE), fingerprinting and sequence analysis of isolated colonies revealed that the cultivable bacteria are affiliated with class *γ-Proteobacteria*, *Bacteroidia*, *Actinobacteria*, *Clostridia* and *β-Proteobacteria*. *γ-Proteobacteria* was the major type of anaerobic cultivable bacteria and even the only one type of aerobic cultivable bacteria. Taken together, our results suggest, for the first time, that anaerobic microbiota are dominant in cultivable bacteria in the special anoxia niche of the fermentation chamber from *H*. *parallela* larvae. These bacterial isolates could be a treasure trove for screening lignocellulytic microbes which are essential for the plant biomass digestion of this scarab species.

## Introduction

Most living organisms, especially insects, have developed a symbiotic relationship with microorganisms. Many microbes inhabit insect alimentary canals, contributing to host food digestion, the production of pheromones, nutrient biosynthesis, longevity, resistance against illness and detoxification [1–3]. The symbiont microbiota may substantially affect various ecological characteristics of insects, especially herbivorous insects (such as the pea aphid), including tolerance to high temperature, resistance to natural enemies, resistance to pathogenic fungi and broadening of food plant range [4–7], as well as the ability of termites to thrive solely on dead plant mass [8]. Even in some cases, the pest status of the host insect was determined by the symbiont bacteria [9].

Scarab beetle (Scarabaeidae) larvae are prevailing pests of grassland and farm soils. They can decay the plant materials, including roots and other low nutritional organic matter [10]. These lignocellulose-rich diets are digested in the scarab beetle’s distinctive intestinal tract typically by lignocellulytic degradation in the hindgut with pretreatment in the alkaline midgut by the solubilizing part of the lignocellulosic materials [11]. The digestion efficiency of plant fibers is up to 65%, and intestinal microorganisms are considered to be essential for this process [10]. Several previous studies revealed that the alimentary tract of scarab beetles contains a dense population of flagellate protozoa and bacteria, and some of these microbes can decompose plant fibers, which are likely involved in the digestion of these herbivorous hosts [12–14].

As the major location for digestion of plant materials, the hindgut of scarab beetle larvae (especially from the pyloric sphincter to the rectum) is an enlarged, distinctive chamber-like structure, which has been considered analogous to the rumen of higher mammals and named the fermentation chamber [10]. This characteristic chamber is lined with cuticle and covered with distinctive lobe-like structures, and it contains many micro-organisms, which play a very important role in the host’s digestion of plant biomass [15]. Zhang and Jackson (2008) revealed that a stable bacterial community is present in the hindgut (fermentation chamber) of *Costelytra zealandica*, and large numbers of bacteria are held in the lobes of the hindgut wall [16]. In *Pachnoda ephippiata* larvae, the accumulation of microbial fermentation products is present in the intestinal tract which is important for the transformation and mineralization of organic matter during gut passage [17]. The fermentation chamber is a typically anaerobic environment and contains a large number of obligate and facultative anaerobic bacteria, which may be critical for (hemi) cellulolytic function [11, 18]. However, the communities of these anaerobic bacteria are still largely unknown.

In China, the larvae of the scarab beetle, *Holotrichia parallela*, causes significant damage to crops by feeding on roots [19]. They possess a typical hindgut fermentation chamber with a lobe-like structure, which is populated by bacteria and covered with cuticular intima [15]. Huang *et al*. (2013) reported that intestinal bacterial communities of *H*. *parallela* larvae changed in response to environmental heterogeneity and host physiological variation to meet the host’s ecological needs and physiological demands [20]. Recently, several aerobic (hemi)cellulytic bacterial strains, as well as endo-xylanase and beta-xylosidase have been reported from the hindgut bacteria of this scarab pest [21, 22]. Currently, however, the anaerobic bacteria have not been studied in this anoxic niche of the fermentation chamber in *H*. *parallela* larvae.

In the current study, culturing of anaerobic and aerobic bacteria associated with the fermentation chamber was counted and isolated. Then denaturing gradient gel electrophoresis analysis of 16S rRNA amplicons (PCR-DGGE) and sequence analysis were used to study the anaerobic and aerobic bacteria communities in the fermentation chambers of *H*. *parallela* larvae. Our results indicate that the anaerobes are prominent in cultivable bacteria of the fermentation chamber in *H*. *parallela* larvae and possess a unique community as compared to aerobes.

## Materials and methods

### Insect rearing and fermentation chamber dissection

*H*. *parallela* larvae were collected from Shandong Province, China. Individual larvae were maintained in a glass jar with local soil (with potato slices as food). The jars were maintained at 25°C and 70% relative humidity (RH). The potato slices were replaced daily.

Individual *H*. *parallela* larvae were sterilized with 70% ethanol before dissection. Immediately after, the intact fermentation chamber (from the pyloric sphincter to the rectum, including the entire inner contents) was cut off from the gut and carefully removed.

### Direct enumeration of total bacterial cells

The dissected fermentation chamber was pooled in a 1.5 ml Eppendorf tube containing 1.0 ml sterile Ringer’s solution (47 mM NaCl, 183 mM KCl, 10 mM Tris-HCl, pH 6.8). Three different individual fermentation chambers were dissected for each replicates, and the number of bacteria were counted as previously described by Cazemier and Hackstein (1997) [14]. In short, the suspensions were vortexed for 30s and subsequently treated by ultra-sonication (Branson B12, Branson Ultrasonics Corp, Danburg, Canada; tip diameter 3 mm, output 40 W) on ice for 45s. A solution of 4’, 6-diamidino-2-phenylindole (DAPI) (5 μg/ml) was used for bacterial staining. The mixture was incubated at 4°C for 30–60min in the dark and was then vortexed for 30 s. A 5 μl aliquot of each suspension was placed on a slide and covered with a 24 mm × 24 mm cover slip and then imaged by fluorescence microscopy (BX53, Olympus Corporation, Tokyo, Japan) for counting bacterial cells. For each sample, 20 fields were counted and each field was 0.08 mm × 0.08 mm, which was equal to a volume of 5.6 × 10^−5^ μl.

### Bacteria culturing, viable count and isolation

The luminal contents of fermentation chambers of three individual larvae were carefully removed using a sterile syringe and immediately injected into a 1.5 ml sterile Eppendorf tube with 0.5 ml of sterile phosphate-buffered saline (PBS) and mixed well. The samples prepared by this process were designated as the **Content Group (CG)** samples. The remaining tissues of the fermentation chambers were washed 3 times with sterile PBS, transferred to a 1.5 ml sterile Eppendorf tube containing 0.5ml sterile PBS buffer, and designated as **Wall Group (WG)** samples. Then, the CG and WG samples were thoroughly homogenized by glass homogenizers and used in bacterial culturing for clustering analysis of cultivable bacterial communities, cultivable bacterial counting and isolation. Both groups were collected in quadruplicate.

For clustering analysis of cultivable bacterial communities of anaerobic WG and CG, and aerobic WG and CG samples, the homogenates of WG and CG samples were cultured in Gifu anaerobic medium (GAM) broth (Nissui, Tokyo, Japan) under anaerobic condition, and in nutrition broth (composed of beef extract 5g/L, peptone 10 g/L, NaCl 5 g/L and agar 15 g/L, adjusted pH to 7.2, and sterilized at 121°C for 20 mins) under aerobic condition, respectively, at 28°C for 72 h. To form the anaerobic condition, culture columns were placed in an anaerobic jar with an anaerobic package as an oxygen scavenger at 28°C. Then the total DNA of these 4 bacteria community samples were extracted for clustering analysis based on PCR-Denaturing gradient gel electrophoresis (DGGE) profiles of partial 16S rRNA gene fragments.

For a viable count of anaerobic bacteria, homogenates of both the CG and WG samples were serially diluted (10^−1^ to 10^−7^), plated in triplicate on GAM agar (GAM broth with 15 g/L of agar) and incubated at an anaerobic condition at 28°C. The number of colonies was recorded after 72 h and used to calculate the initial inoculum size. For the viable count of aerobic bacteria, the culture medium was replaced with nutrition agar (nutrition broth with 15 g/L of agar), and the culture dishes were placed in a normal aerobic condition at 28°C.

For cultivable bacterial isolation, ten colonies from each appropriate agar plate of anaerobes and aerobes were randomly inoculated into GAM broth under anaerobic condition and NA broth under aerobic condition, respectively. Incubation lasted for 16 h at 28°C and then DNA was extracted for genotype identification by PCR-DGGE analysis of partial 16S rRNA gene fragments.

### DNA extraction

DNA was extracted according to Zhang et al. (2003) [16], with little modification as follows: The bacteria sample was centrifuged (6000 r/min, 4°C) for 1 min and the supernatants were discarded. Immediately after, 1.0 ml of sterile distilled water was added into each tube, and then the tubes were vortexed for 1 min. The mixtures were centrifuged, and the supernatants were discarded. This procedure was repeated 3 times. The pellets were re-suspended in 300 μl high saline DNA extraction buffer (100 mM Tris-HCl, 100 mM EDTA, 100 mM sodium phosphate, 1.5 M sodium chloride, and 1% CTAB; pH 8.0) and were heated on a shaking plate at 250 r/min, 37°C for 30 min. Then 10 μl of proteinase K (20 mg/ml) and 50 μl of 20% SDS solution were added, followed by incubation at 65°C for 2 h. Immediately after, an equal volume of chloroform-isoamyl alcohol-phenol (25:24:1 v/v/v) was added, mixed and centrifuged (13000 r/min, 4°C) for 5 mins. The upper layer solution was transferred into a new 1.5 ml tube, and isopropanol (2/3 volume) and 50 μl of sodium acetate (3 M) were added to precipitate the DNA. The DNA pellets were re-suspended in 100 μl of sterile distilled water and stored at -20°C until use.

### PCR amplification of partial bacterial 16S rRNA gene fragments

F968GC (5’ gc clamp-AACGCGAAGAACCTTAC 3’) and L1401 (CGGTGTGTACAAGACCC) as the forward primer and reverse primer, respectively, were employed for the DGGE analysis with a GC clamp (5’ CGCCCGCCGCGCGCGGCGGGCGGGGCGGGGGCACGGGGGG 3’) attached to the 5’ end of primer F968 (5’ AACGCGAAGAACCTTAC 3’) [23]. The reaction mixture (50 μl) consisted of 1 μl of DNA template, 1 μl of each primer (10μM), 1 μl of dNTPs (10 mM), 5 μl of 10× buffer, 6 μl of MgCl_2_ (25 mM) and 1.25 units of Taq polymerase (TAKARA, Dalian, China). The PCR was performed in a PTC-200 Thermal Cycler (Bio-Rad, Hercules, CA, USA) under the following conditions: initial denaturation at 95°C for 5 min, 35 cycles of elongation (1 min of denaturation at 94°C, 30 s of annealing at 56°C and 30 s of extension at 68°C) and a final extension at 72°C for 10 min. The amplified PCR products were visualized by electrophoresis on a 1.2% agarose gel and then purified for DGGE analysis using the QIAquick PCR purification kit (Qiagen, Hilden, Germany).

### Denaturing gradient gel electrophoresis (DGGE)

The PCR amplicons of partial bacterial 16S rRNA gene fragments were analyzed according to the DGGE techniques from Zhang and Jackson (2008) [16]. Briefly, the purified PCR products from colonies were electrophoresed with a linear denaturing gradient of 30%-70% denaturant on a Detection Code System (Bio-Rad, Hercules, CA, USA) with a constant voltage (85 V) for 16 h at 60°C and then stained with silver nitrate, according to Sanguinetti et al. (1994) [24]. Isolated colonies exhibiting the bands at the same position in the gel were regarded as the same bacterial genotypes.

### Cloning and sequencing of 16S rRNA gene fragments

Only one PCR amplicon of the unique genotype was ligated into the pMD-18 vector by using a TAKARA cloning kit (TAKARA, Dalian, China) and introduced into *Escherichia coli* DH5α cells. The plasmid insertions were confirmed by PCR amplification with the 16S rRNA primer F968GC and the M13R (5’ CGCCAGGGTTTTCCCAGTCACGAC 3’). These PCR products were run on a DGGE gel to measure the electrophoretic mobility of insertions, and clones with the correct insertion were sequenced in both directions.

### Data analysis

One-way analysis of variance (ANOVA) was used to compare the means of the viable plate counts of anaerobic (WG & CG) and aerobic (WG &CG) bacteria, followed by a post hoc Duncan’s test for individual group comparisons using the statistical software SPSS 10.0 (Statistical Package for Social Science, Chicago, USA). The similarity between bacterial communities was represented by cluster dendrograms based on the analysis of the Euclidean squared distance coefficient, followed by unweighted pair-group method analysis (UPGMA). All 16S rRNA gene sequence data were edited with DNAMAN for Windows (version 6, Lynnon BioSoft, Quebec, Canada), and taxonomic annotation was performed by Ribosomal Database Project (RDP) classifier analysis (RDP Release 11, https://rdp.cme.msu.edu/classifier/classifier.jsp), which provides taxonomic assignments from domain to genus with confidence estimates [25]. The sequences were compared to public databases using BLASTn (https://blast.ncbi.nlm.nih.gov/Blast.cgi). The most closely related sequences were retrieved and added for alignments. By using MEGA (version 5), the alignments were analyzed by the Maximum likelihood method, and then the phylogenetic tree was constructed by neighbor-joining (NJ) algorithms [26].

### Nucleotide sequence accession numbers

The accession numbers of submitted sequences allotted from GenBank nucleotide sequence database are FJ533232 to FJ533269.

## Results

### Direct enumeration and viable count of bacterial cells in the fermentation chamber of *H*. *parallela* larvae

The enumeration results, shown in Table 1, indicated that a total of 1.67±0.09×10^10^ bacterial cells/gut in fermentation chamber were directly enumerated by the DAPI staining method, while 15.36±1.41×10^7^ cfu/gut of bacteria were found by viable counts under both anaerobic and aerobic conditions. The bacterial count was only 0.92% of the total bacteria, which indicated that a very low number of bacteria in the fermentation chamber were successfully cultured under normal culturing conditions.

**Table 1:** Viable counts of bacterial cells in the fermentation chambers of *Holotrichia paralella* larvae.

Culture conditions	Samples	Viable counts (Means ± Std. Error) (cfu/gut)	Percentage (%)
Aerobic	Wall Group(WG)	3.68 ± 0.63 × 10^7^ a*	23.96
	Contents Group(CG)	1.47 ± 0.27 × 10^7^ a	9.57
Anaerobic	Wall Group(WG)	8.67 ± 1.42 × 10^7^ b	56.45
	Contents Group(CG)	1.55 ± 0.38 × 10^7^ a	10.09
Total		15.36 ± 1.41 × 10^7^	

*Means followed by the same letter in each column were not significantly different (*P* <0.05) by post hoc Duncan’s test for individual group comparisons after One-way analysis of variance (ANOVA).

The number of cultivable bacteria from the WG samples (12.35×10^7^ cfu/gut) was 4.09 times larger than the CG samples (3.02×10^7^ cfu/gut). This indicated that the cultivable bacteria in the larval fermentation chamber were more apt to adhere to the chamber wall. The anaerobic viable counts (10.22×10^7^ cfu/gut) were 1.98 times larger than aerobic viable counts (5.15×10^7^ cfu/gut), which revealed that the condition of the fermentation chamber was more suitable for anaerobic bacteria. Moreover, in the WG samples, there were 2.36 times more anaerobic bacteria (8.67±1.42×10^7^ cfu/gut) than their aerobic counterparts (3.68±0.63×10^7^ cfu/gut), while the ratio of anaerobic bacteria (1.55±0.38×10^7^ cfu/gut) to aerobic counterparts (1.47±0.27×10^7^ cfu/gut) in the CG samples was only 1.05. These results indicate that, compared to the chamber contents, the cultivable bacteria, especially the anaerobic cultivable bacteria, tend to distribute associated with the fermentation chamber wall.

### DGGE profiles of PCR-amplified bacterial partial 16S rRNA gene fragments

**Fig 1:**
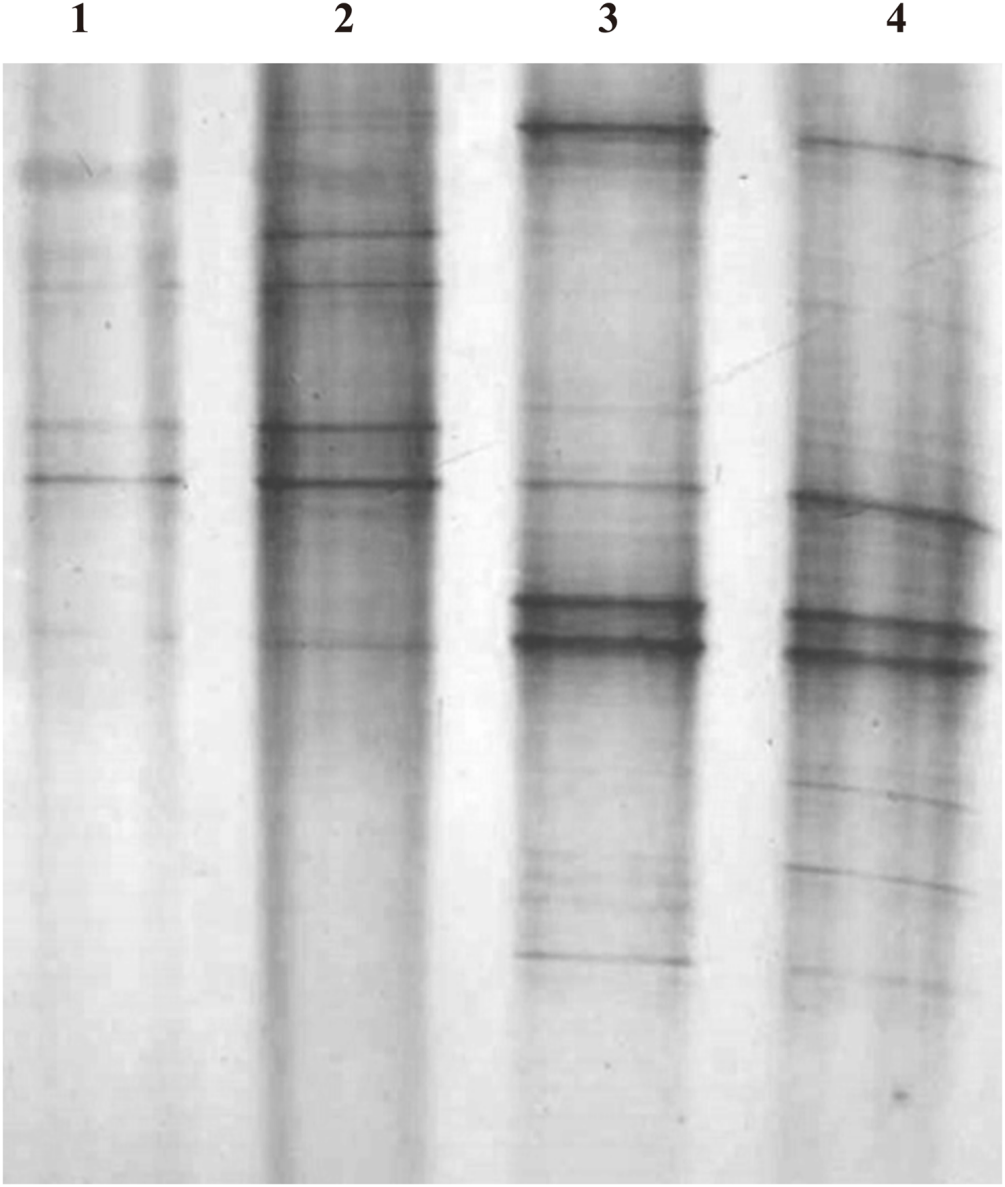
Denaturing gradient gel electrophoresis (DGGE) profiles of polymerase chain reaction (PCR)-amplified bacterial partial 16S rRNA gene fragments from different bacterial communities in the fermentation chamber of *Holotrichia parallela* larvae. (1) Aerobic bacteria in fermentation chamber contents; (2) Aerobic bacteria from fermentation chamber wall; (3) Anaerobic bacteria in fermentation chamber contents; (4) Anaerobic bacteria from fermentation chamber wall.

**Fig 2:**
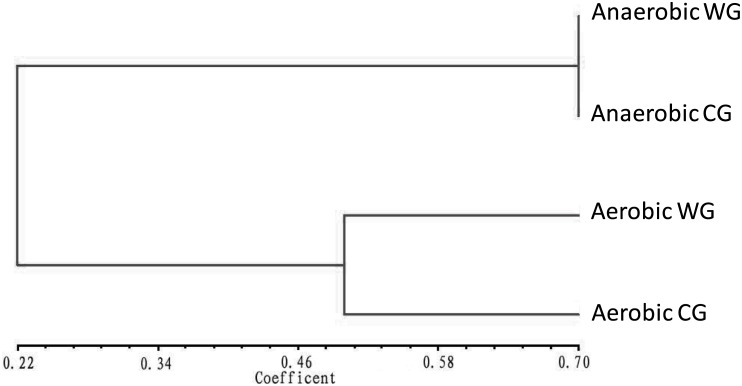
Dendrogram of DGGE profiles of PCR-amplified partial 16S rRNA gene fragments from different bacterial communities in the fermentation chamber of *H*. *parallela* larvae. The clustering dendrogram was constructed based on the Euclidean squared distance coefficient, which was followed by an unweighted pair-group method analysis (UPGAMA) method. The value of similarity level was indicated on the bottom line. WG: group of fermentation chamber wall. CG: group of fermentation chamber content.

The DGGE profiles of PCR-amplified bacterial partial 16S rRNA gene fragments of 4 bacterial communities (anaerobic WG and CG, and aerobic WG and CG) were presented in Fig 1 and classified into two groups, i.e., anaerobic and aerobic groups with 0.675 and 0.465 of clustering distance value, respectively (Fig 2). This indicated that the anaerobic bacterial communities of WG and CG are more consistent in bacteria composition than aerobic bacterial communities of WG and CG.

**Fig 3:**
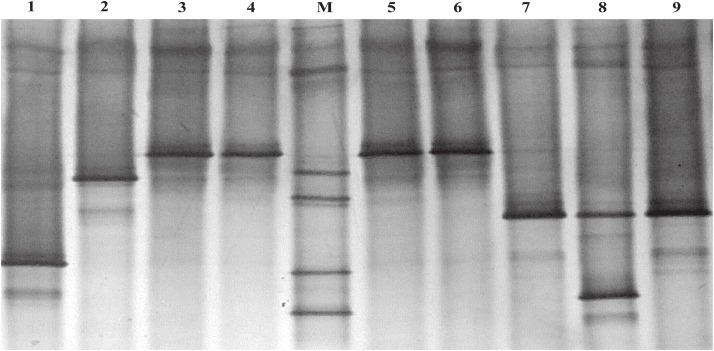
DGGE profile of PCR-amplified partial 16S rRNA gene fragments of single bacterial colony from fermentation chambers of *H*. *parallela* larvae. Lane 1, 2 and 8 showed three unique band profiles of 16S rRNA PCR fragment, which represented three unique genotypes. Lane 3–6 showed the same band profile, which had been classified into one unique genotype, and so did lane 7 and 9. The DNA marker (M) consisted of PCR-amplified partial 16S rRNA gene fragments of seven bacterial clones which showed different electrophoretic mobility in DGGE gel.

By using plate cultivation-dependent microbiological methods, a total of 491 cultivable bacterial colonies were isolated from the fermentation chamber of *H*. *parallela* larval. The DGGE profiles of PCR-amplified bacterial partial 16S rRNA gene fragments of each colony isolation revealed 38 unique bands profiles. Each unique band profile represented a unique genotype of bacteria, and the bacterial colonies with the same band profile were classified into one unique genotype (Fig 3). So these 491 cultivable bacterial colonies were classified into 38 genotypes, 16 and 22 genotypes of which were identified from anaerobic and aerobic bacteria, respectively (Tables 2 and 3).

**Table 2:** Anaerobic cultivable bacterial genotypes in the fermentation chambers of *H*. *paralella* larvae based on 16S rRNA gene sequence analysis.

Clone code	location	Accession No.	Class ^a^	Family ^a^	Genus ^a^	Closest known species (Accession No.)	Similarity (%)	Number of isolates	Percentage of total isolates
B39	Wall Group (WG)	FJ533243	*γ-Proteobacteria* [100%]	*Enterobacteriaceae* [100%]	*Klebsiella* [53%]	Uncultured bacterium clone 34M2 (DQ079820)	99	69	33.17%
B22		FJ533241	*γ-Proteobacteria* [100%]	*Enterobacteriaceae* [100%]	*Enterobacter* [87%]	*Enterobacter cloacae* strain EB89 (FJ194527)	99	16	7.69%
B21		FJ533247	*Bacteroidia* *[61%]*	*Porphyromonadaceae* *[61%]*	*Petrimonas* [19%]	Uncultured bacterium (EU773762)	91	11	5.29%
B30		FJ533242	*γ-Proteobacteria* [100%]	*Enterobacteriaceae* [100%]	*Citrobacter* [91%]	*Citrobacter farmeri* (AF025371)	99	9	4.33%
B31		FJ533246	*Actinobacteria* [54%]	*Actinomycetales* [54%]	*Micrococcineae* [54%]	Uncultured *Rheinheimera* sp. clone F5OHPNU07ID24Q (HQ091615)	94	8	3.85%
B15		FJ533240	*γ-Proteobacteria* [100%]	*Enterobacteriaceae* [100%]	*Citrobacter* [57%]	Uncultured bacterium clone C80 (FJ356046)	99	7	3.37%
B1		FJ533244	*Clostridia* [100%]	*Peptostreptococcaceae* [100%]	*Clostridium XI* [98%]	*Clostridium venationis* (EU089966)	98	7	3.37%
B12		FJ533245	*β-Proteobacteria* [100%]	unspecified *Burkholderiales* family [100%]	*Aquabacterium* [100%]	Uncultured bacterium clone MB14 (FJ204069)	99	5	2.40%
Q8	Content Group (CG)	FJ533239	*γ-Proteobacteria* [100%]	*Enterobacteriaceae* [100%]	*Enterobacter* [64%]	*Klebsiella pneumoniae* subsp. *Ozaenae* (AF130982)	98	15	7.21%
Q29		FJ533237	*γ-Proteobacteria* [100%]	*Enterobacteriaceae* [100%]	*Klebsiella* [46%]	Uncultured bacterium clone 34M2 (DQ079820)	99	12	5.77%
Q2		FJ533233	*Bacteroidia* [100%]	*Porphyromonadaceae* [100%]	*Dysgonomonas* [100%]	*Dysgonomonas sp*. TW5-36(KR822476)	98	12	5.77%
Q13		FJ533235	*γ-Proteobacteria* [100%]	*Enterobacteriaceae* [100%]	*Enterobacter* [47%]	Uncultured bacterium clone ZS66 (KU241008)	99	9	4.33%
Q9		FJ533234	*Bacteroidia* [100%]	*Porphyromonadaceae* [100%]	*Dysgonomonas* [100%]	Uncultured bacterium clone B68(JN006180)	98	9	4.33%
Q33		FJ533232	*Bacteroidia* [100%]	*Porphyromonadaceae* [100%]	*Dysgonomonas* [100%]	Uncultured *Bacteroidetes* bacterium (AB522123)	99	8	3.85%
Q20		FJ533236	*Bacteroidia* [100%]	*Porphyromonadaceae* [100%]	*Dysgonomonas* [100%]	Uncultured bacterium clone B56 (JN006178)	98	7	3.37%
Q21		FJ533238	*γ-Proteobacteria* [100%]	*Enterobacteriaceae* [100%]	*Klebsiella* [78%]	Uncultured bacterium clone 12M2 (DQ079819)	98	4	1.92%

^***a***:^ The taxonomic affiliation of each bacterial genotype was estimated in class, family and genus levels by using the web-based Ribosomal Database Project (RDP) classifier analysis (RDP Release 11, https://rdp.cme.msu.edu/classifier/classifier.jsp). The numbers in the brackets indicated the confidence (bootstrap) values of RDP classifier analysis.

**Table 3:** Aerobic cultivable bacterial genotypes in the fermentation chambers of *H*. *paralella* larvae based on 16S rRNA gene sequence analysis.

Clone code	location	Accession No.	Class ^a^	Family ^a^	Genus ^a^	Closest known species (Accession No.)	Similarity (%)	Number of isolates	Percentage of total isolates
B267	Wall Group (WG)	FJ533267	*γ-Proteobacteria* [100%]	*Enterobacteriaceae* [100%]	*Enterobacter* [99%]	Uncultured *Enterobacter* sp. clone LR148 (HM597909)	99	63	22.26%
B236		FJ533265	*γ-Proteobacteria* [100%]	*Enterobacteriaceae* [100%]	*Enterobacter* [59%]	Uncultured bacterium clone PB2_aai21d08 (EU777815)	98	14	4.95%
B221		FJ533262	*γ-Proteobacteria* [100%]	*Enterobacteriaceae* [99%]	*Trabulsiella* [66%]	Uncultured bacterium clone PCC-4 (EF608526)	95	12	4.24%
B201		FJ533259	*γ-Proteobacteria* [100%]	*Enterobacteriaceae* [100%]	*Enterobacter* [89%]	Uncultured bacterium clone RDX 15 (EU907879)	99	10	3.53%
B272		FJ533268	*γ-Proteobacteria* [100%]	*Enterobacteriaceae* [100%]	*Enterobacter* [69%]	*Enterobacter* sp. Y4 (DQ821728)	98	7	2.47%
B206		FJ533260	*γ-Proteobacteria* [100%]	*Enterobacteriaceae* [100%]	*Enterobacter* [92%]	*Enterobacter ludwigii* strain YS2 (KY887767)	99	6	2.12%
B234		FJ533263	*γ-Proteobacteria* [100%]	*Enterobacteriaceae* [100%]	*Klebsiella* [50%]	Uncultured bacterium clone EHB-PS1001 (KU978368)	99	5	1.77%
B210		FJ533261	*γ-Proteobacteria* [100%]	*Enterobacteriaceae* [100%]	*Enterobacter* [64%]	Uncultured bacterium clone EHB-PS0427 (KU978324)	99	5	1.77%
B277		FJ533269	*γ-Proteobacteria* [100%]	*Enterobacteriaceae* [100%]	*Citrobacter* [91%]	*Citrobacter* sp. KSL 4–091 (FJ481101)	99	4	1.41%
B247		FJ533266	*γ-Proteobacteria* [100%]	*Enterobacteriaceae* [100%]	*Citrobacter* [93%]	*Grimontella senegalensis* (AY217653)	99	2	0.71%
B235		FJ533264	*γ-Proteobacteria* [100%]	*Enterobacteriaceae* [100%]	*Klebsiella* [24%]	Uncultured *Enterobacteriaceae* bacterium clone SKF016 (JF733260)	96	2	0.71%
Q151	Content Group (CG)	FJ533258	*γ-Proteobacteria* [100%]	*Enterobacteriaceae* [100%]	*Enterobacter* [75%]	*Enterobacter cancerogenus* (FM210030)	99	97	34.28%
Q110		FJ533248	*γ-Proteobacteria* [100%]	*Enterobacteriaceae* [100%]	*Klebsiella* [95%]	Uncultured bacterium clone SC24 (JF964694)	98	11	3.89%
Q164		FJ533256	*γ-Proteobacteria* [100%]	*Enterobacteriaceae* [100%]	*Klebsiella* [83%]	Uncultured *Klebsiella* sp. clone LSSR193 (HM597960)	99	9	3.18%
Q159		FJ533257	*γ-Proteobacteria* [100%]	*Enterobacteriaceae* [100%]	*Enterobacter* [93%]	*Enterobacter cloacae* strain XJU-PA-7 (EU733519)	99	8	2.83%
Q175		FJ533254	*γ-Proteobacteria* [100%]	*Enterobacteriaceae* [100%]	*Klebsiella* [81%]	*Klebsiella* sp. Gc-7-c (FJ159440)	99	8	2.83%
Q147		FJ533251	*γ-Proteobacteria* [100%]	*Enterobacteriaceae* [100%]	*Enterobacter* [74%]	Uncultured bacterium clone A2_806 (KR304480)	98	7	2.47%
Q189		FJ533253	*γ-Proteobacteria* [100%]	*Enterobacteriaceae* [100%]	*Enterobacter* [48%]	Uncultured bacterium clone NYSYF111 (EU879477)	98	4	1.41%
Q135		FJ533249	*γ-Proteobacteria* [100%]	*Enterobacteriaceae* [90%]	*Cedecea* [27%]	Uncultured bacterium clone JH-YT47 (EF033238)	98	3	1.06%
Q136		FJ533250	*γ-Proteobacteria* [100%]	*Enterobacteriaceae* [100%]	*Pseudocitrobacter* [81%]	Uncultured *Enterobacteriaceae* bacterium clone SKF016 (JF733260)	95	3	1.06%
Q172		FJ533255	*γ-Proteobacteria* [100%]	*Enterobacteriaceae* [100%]	*Klebsiella* [94%]	Uncultured bacterium (FM865635)	99	2	0.71%
Q186		FJ533252	*γ-Proteobacteria* [100%]	*Enterobacteriaceae* [100%]	*Klebsiella* [97%]	*Klebsiella pneumoniae* strain FIUMS1 (FJ436718)	99	1	0.35%

^***a***:^ The taxonomic affiliation of each bacterial genotype was estimated in class, family and genus levels by using the web-based RDP classifier analysis (RDP Release 11, https://rdp.cme.msu.edu/classifier/classifier.jsp). The numbers in the brackets indicated the confidence (bootstrap) values of RDP classifier analysis.

### Communities of the cultivable bacteria in the fermentation chamber of *H*. *parallela* larval

**Fig 4:**
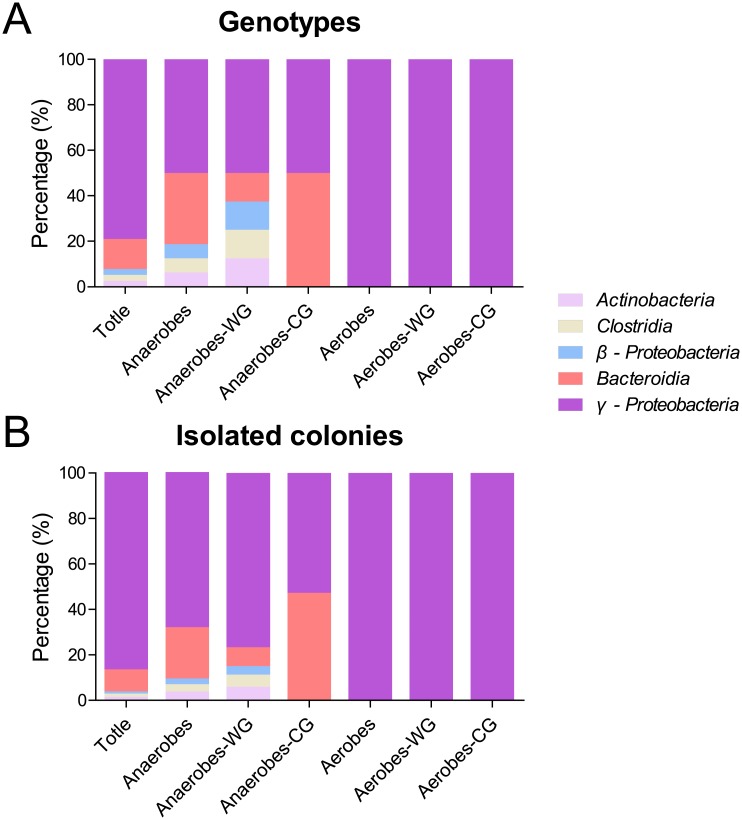
Relative abundance of different bacterial phylogenetic groups in the fermentation chamber of *H*. *parallela* larvae. These genotypes or isolates were sorted into 7 different bacterial groups, including total bacteria, anaerobic bacteria, anaerobic bacteria from fermentation wall (Anaerobes-WG), anaerobic bacteria in fermentation content (Anaerobes-CG), aerobic bacteria from fermentation wall (Aerobes-WG) and aerobic bacteria in fermentation content (Aerobes-CG). (A) The percentage distribution was calculated based on the relative abundance in all genotypes of each sample; (B) The percentage distribution was calculated on the basis of relative abundance in total isolated colonies of each sample.

By using the partial sequences of 16S rRNA gene fragments, 38 genotypes were identified by the RDP classifier analysis which showed that all genotypes were affiliated with five classes: *γ-Proteobacteria* (including family *Enterobacteriaceae*), *Bacteroidia* (*Porphyromonadaceae*), *Actinobacteria* (*Actinomycetales*), *Clostridia* (*Peptostreptococcaceae*) and *β-Proteobacteria* (including unspecified *Burkholderiales* family). These five classes included 78.95%, 13.16%, 2.63%, 2.63% and 2.63% of the genotypes, and 86.36%, 9.58%, 1.63%, 1.43% and 1.02% of the isolated colonies of cultivable bacteria, respectively (Fig 4). This result indicates that most of the cultivable bacteria were classified into class *γ-Proteobacteria* in both strain species and counts.

#### (a) Community of the anaerobic cultivable bacteria

**Fig 5:**
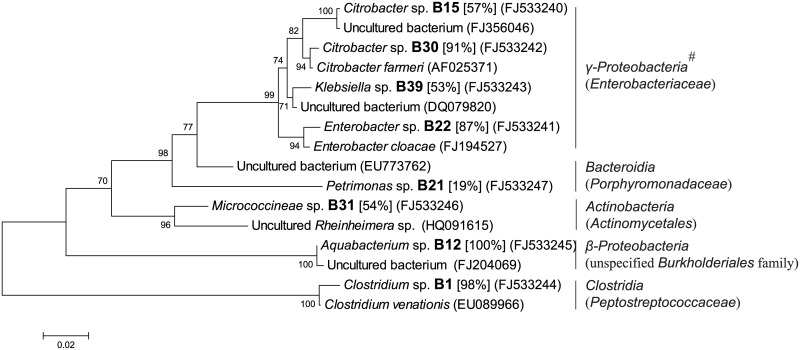
Phylogenetic tree based on partial 16S rRNA sequence of unique genotypes of anaerobic bacterial colonies from fermentation chamber wall (WG) of *H*. *parallela* larvae. The tree was constructed using maximum likelihood criteria and the neighbour-joining method. GenBank accession numbers of all sequences were indicated in parentheses. Each genotype name of isolated cultivable clone was displayed in bold font with genus name predicted by RDP classifier analysis (confidence value was presented in bracket). Bar represents 0.02 substitutions per site. #: bacterial classes (families in brackets) from the RDP II database.

**Fig 6:**
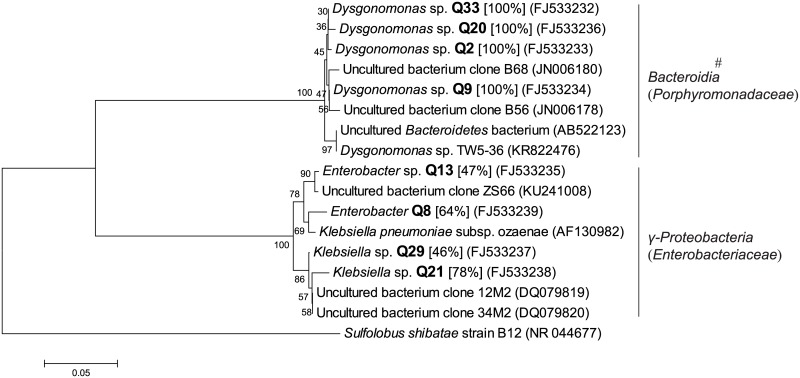
Phylogenetic tree based on partial 16S rRNA sequence of unique genotypes of anaerobic bacterial colonies in fermentation chamber contents (CG) of *H*. *parallela* larvae. The tree was constructed using maximum likelihood criteria and the neighbour-joining method. GenBank accession numbers of all sequences were indicated in parentheses. Each genotype name of isolated cultivable clone was displayed in bold font with genus name predicted by RDP classifier analysis (confidence value was presented in bracket). *Sulfolobus shibatae* strain B12 (NR_044677) was used as outgroup. Bar represents 0.05 substitutions per site. #: bacterial classes (families in brackets) from the RDP II database.

Under anaerobic culture conditions, 208 colonies were isolated and classified into 16 genotypes by DGGE profiles of PCR-amplified partial 16S rRNA gene fragments. Phylogenetic analysis of partial 16S rRNA sequence revealed that these genotypes were affiliated with five classes: *γ-Proteobacteria* (including family *Enterobacteriaceae*), *Bacteroidia* (*Porphyromonadaceae*), *Actinobacteria* (*Actinomycetales*), *Clostridia* (*Peptostreptococcaceae*) and *β-Proteobacteria* (including unspecified *Burkholderiales* family) (Figs 5 and 6). All concerning classes included 50.00%, 31.25%, 6.25%, 6.25% and 6.25% of the genotypes, as well as 67.79%, 22.61%, 3.85%, 3.37% and 2.40% of the isolated colonies of cultivable anaerobic bacteria, respectively (Fig 4).

Among these colonies of anaerobes, 132 colonies were isolated from the WG samples and classified into 8 genotypes. These genotypes were distributed among five classes: *γ-Proteobacteria* (including family *Enterobacteriaceae*), *Bacteroidia* (*Porphyromonadaceae*), *Actinobacteria* (*Actinomycetales*), *Clostridia* (*Peptostreptococcaceae*) and *β-Proteobacteria* (including unspecified *Burkholderiales* family) (Fig 5), which included 50.00%, 12.50%, 12.50%, 12.50% and 12.50% of the genotypes, as well as 76.52%, 8.33%, 6.06%, 5.30% and 3.79% of the isolated colonies of cultivable bacteria from anaerobic WG samples, respectively (Fig 4).

Differing from anaerobes of WG, 76 colonies of anaerobic bacteria were isolated from the CG samples, which were classified into 8 genotypes. These genotypes were only distributed in *γ-Proteobacteria* (including family *Enterobacteriaceae*) and *Bacteroidia* (*Porphyromonadaceae*) (Fig 6), which had 50% and 50% of the genotypes, as well as 52.63% and 47.37% of the isolated colonies of cultivable bacteria from anaerobic CG samples, respectively (Fig 4).

#### (b) Community of the aerobic cultivable bacteria in the fermentation chamber of *H*. *parallela* larval

**Fig 7:**
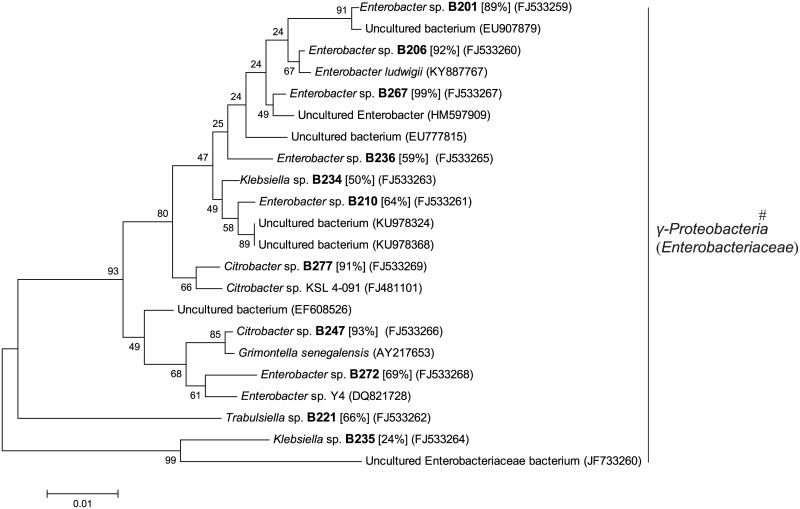
Phylogenetic tree based on partial 16S rRNA sequence of unique genotypes of aerobic bacterial colonies from fermentation chamber wall (WG) of *H*. *parallela* larvae. The tree was constructed using maximum likelihood criteria and the neighbour-joining method. GenBank accession numbers of all sequences were indicated in parentheses. Each genotype name of isolated cultivable clone was displayed in bold font with genus name predicted by RDP classifier analysis (confidence value was presented in bracket). Bar represents 0.01 substitutions per site. #: bacterial classes (families in brackets) from the RDP II database.

**Fig 8:**
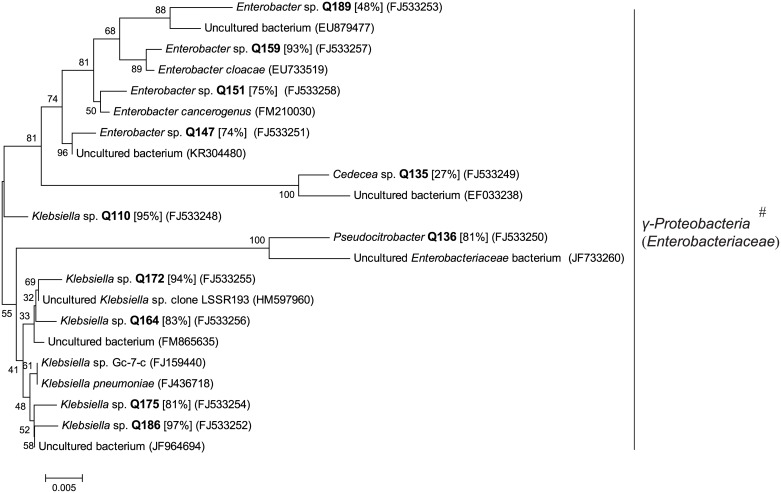
Phylogenetic tree based on partial 16S rRNA sequence of unique genotypes of aerobic bacterial colonies in fermentation chamber contents (CG) of *H*. *parallela* larvae. The tree was constructed using maximum likelihood criteria and the neighbor-joining method. GenBank accession numbers of all sequences were indicated in parentheses. Each genotype name of isolated cultivable clone was displayed in bold font with genus name predicted by RDP classifier analysis (confidence value was presented in bracket). Bar represents 0.005 substitutions per site. #: bacterial classes (families in brackets) from the RDP II database.

Under aerobic culturing conditions, 283 colonies were isolated and classified into 22 genotypes by DGGE profiles of PCR-amplified partial 16S rRNA gene fragments. Phylogenetic analysis of partial 16S rRNA gene sequences demonstrated that these genotypes were all affiliated with one class, *γ-Proteobacteria* (including family *Enterobacteriaceae*) (Figs 4, 7 and 8).

Among these colonies of aerobes, 130 colonies and 153 colonies were isolated from the WG and CG samples correspondingly, and classified into 11 genotypes and 11 genotypes, respectively. These genotypes were affiliated with one class, *γ-Proteobacteria* (including family *Enterobacteriaceae*).

## Discussion

The present results demonstrated that only 0.92% of the total bacteria in the fermentation chamber of *H*. *parallela* could be cultivated, which is similar to the conclusions of Ward et al. (1992), Kepner et al. (1994) and Amann et al. (1995) [27–29]. All previous reported studies indicate that traditional cultivation-dependent microbiological methods for the analysis of bacterial diversity are only capable of detecting very few bacteria from any particular environmental sample. Currently, there are still many unknown uncultivable bacteria, due to the limited techniques of the culture.

In the present research, PCR-DGGE analysis based on traditional viable plate count and clone isolation were selected to investigate the cultivable anaerobic and aerobic bacterial community of the fermentation chamber, including lumen content and wall, of *H*. *parallela*. Although a high throughput sequencing strategy based on a cultivation-independent method has been widely applied in constructing the whole profiles of gut microbiotic communities in diverse insect species, including *H*. *parallela* [30, 31], the anaerobic microbiota, which should be dominant in an extremely anaerobic niche of fermentation chambers in grass grubs [13], were largely unknown. To identify the anaerobic and aerobic microbes separately, traditional cultivation-dependent methods under anaerobic and aerobic conditions, respectively, are still necessary. Through cultivation-dependent method, isolated cultivable bacterial clones are limited in hundreds or thousands. So PCR-DGGE method is suitable for screening the varied genotypes of these clones for molecular microbial ecology because of its high sensitivity and efficiency with limited cost [32–35], which has been applied in our present study on the cultivable anaerobic and aerobic bacterial communities of the fermentation chamber in *H*. *parallela* separately through cultivation-dependent methods.

Our research revealed that most of the cultivable bacteria in the fermentation chambers were anaerobic species and adhered to the chamber wall. Compared to the chamber content, most cultivable bacteria were associated with the chamber wall (4.09-fold higher) (Table 1). Furthermore, in the chamber wall, most of the cultivable bacteria were identified as anaerobes (2.36-fold higher than aerobes, Table 1). These results suggest that this special niche of the fermentation chamber wall is much more suitable for anaerobic bacteria. This could be explained by the unique anaerobic environment of the fermentation chamber of *H*. *parallela*, a unique environment that is greatly anaerobic and suitable for microbiota colonization. It even forms a special lobe-like structure [15]. As a specialized structure developed along the evolution of scarab beetles, the fermentation chambers are critical for these herbivores for the digestion of lignocellulose-rich diets through symbiotic associations with lignocellulolytic microbes [10, 17]. Our findings provide evidence that these symbionts have adapted to the anoxic environment of the fermentation chamber, especially in the wall structure (such as the lobe-like structure) [15], and are speculated to be important in digesting lignocellulose-rich diets in the scarabs. This implies that the anaerobes from the fermentation chamber could be a treasure trove for discovering valuable microorganisms with lignocellulytic activity for the biomass energy industry. This warrants further studies in the future, And in fact, the anaerobic colonies isolated in the present studies are currently being screened for lignocellulytic activity.

In our results, all genotypes of cultivable bacteria from the fermentation chamber of *H*. *parallela* larvae were classified into five classes, including *γ-Proteobacteria*, *Bacteroidia*, *Actinobacteria*, *Clostridia* and *β-Proteobacteria*, which have also been reported as major components of the hindgut microbiota of *H*. *parallela* larvae through cultivation-independent investigations [20, 31]. One prominent feature of the cultivable microbiota in the present study is the dominance of genotypes affiliated with the class *γ-Proteobacteria* (including family *Enterobacteriaceae*) (Fig 4). Furthermore, all genotypes of cultivable aerobes were identified as only *γ-Proteobacteria* (*Enterobacteriaceae*). A similar cultivable bacterial community has also been reported in another grass grub species, *Melolontha hippocastani*, which indicated the extreme domination of *γ-Proteobacteria* (99.5% of *Enterobacteriaceae* species including *Serratia* spp. and *Citrobacter* sp.) in the hindgut homogenate of third-instar larvae through similar cultivation-dependent method [36]. This is quite different from previous research, which reported the phylum *Firmicutes*, class *Clostridia* as the prominent species of microbiota, either in composition of genotypes (or operational taxonomic units, OTUs), or either in *H*. *parallela* or other grass scarab species [16, 20, 37]. This variation could be induced by the difference of cultivation-dependent and independent methods for bacterial 16s rRNA sequencing, the former of which represented very low proportion of the bacterial community (low to 1%) and may enrich the bacterial species adapted to grow on the provided media. This enrichment of *Proteobacteria* caused by varied investigating methods has also been found in the turtle ant, *Cephalotes varians*, which depicted increased relative abundance of *Proteobacteria* in a gut bacterial community by a cultivation-dependent method [viable counting on lysogeny broth (LB) agar plates] when compared to a cultivation-independent method through 454 pyrosequencing [38]. In addition, the varied grass grub samples used in our present study may also affect the bacterial community structure, which could be influenced by environmental heterogeneity (such as geographic region, food sources, soil characteristics, etc.) as previously described in *H*. *parallela* [20].

In the current research, for the first time, the anaerobic cultivable bacterial community of the fermentation chamber, which has been largely uncharacterized in *H*. *parallela*, an important agricultural pest in China, was reported using an anaerobic method. A comparison between cultivable anaerobic and aerobic bacteria, revealed that anaerobes are more dominant, with fewer genotypes (16 genotypes in anaerobes *vs*. 22 in aerobes), but larger taxonomic range (3 classes in anaerobes *vs*. 1 in aerobes). It is a remarkable fact that most of the cultivable anaerobes are identified as phylum *Proteobacteria*, including *γ-Proteobacteria* (50.00% of genotypes and 67.79% of colony counts) and *β-Proteobacteria* (6.25% of genotypes and 2.40% of colony counts), additionally all cultivable aerobes are identified as phylum *Proteobacteria*, (*γ-Proteobacteria*), which are universally distributed in animal guts and environments [3, 39]. In fact, we have identified several strains affiliating with *Proteobacteria* with high activities of cellulase and xylanase from the isolates obtained in our present research (data not shown), and the cellulase gene family were most associated with the phylum *Proteobacteria* in the hindgut of *H*. *parallela* [40]. In addition, for this specific grass grub, the cellulose induced cultivation with ligonocellulose-rich diet (containing rice straw and filter paper strip) also led to a significant enrichment of phyla *Proteobacteria* [31]. In some special microbiomes with lignocellulolytic function, such as the fungus garden microbiome of leaf-cutter ant and compost microbiome, the *Proteobacteria* bacteria are also the major components, which are speculated to play an important role in the degradation and digestion of plant mass [41, 42]. In addition, some *Proteobacteria* (such as *Klebsiella variicola* At-22 and *Pantoea* sp. At-9b from fungus garden microbiome of leaf-cutter ant, and *Saccharophagus degradans* strain 2-40^T^ from marine areas) have been associated with extreme lignocellulolytic activity [41, 43]. These all imply the dominant effect of *Proteobacteria* in lignocellulose-rich diets digestion in the fermentation chamber of *H*. *parallela*, which led to further screening of ligonocellulolytic bacteria.

In addition to the function of plant biomass degradation, these cultivable bacteria (such as *Proteobacteria* anaerobes) also play an important role in other bio-functions, such as oxygen depletion and nutrition supply. In the Formosan termite, intestinal *Serratia marcescens* is a facultative anaerobe and aids in consuming oxygen at the periphery of the insect stomach to maintain a habitable gut for the strict anaerobes that digest cellulose [44]. Lauzon et al. (2000) reported that *Enterobacter* spp. assists nitrogen recycling in *Rhagoletis pomonella* (Walsh) by producing uricases [45]. In addition, some *Enterobacteriaceae*, such as the genera *Citrobacter*, *Enterobacter*, and *Klebsiella*, also have the ability to ferment glucose to aid host insect nutrition [46].

In conclusion, we have characterized the cultivable bacterial community in the fermentation chambers of *H*. *parallela* larvae by cultivation-dependent PCR-DGGE and sequence analysis. The viable count results showed that only a small portion of bacteria could be cultivable under anaerobic and aerobic conditions. Compared to the aerobic bacteria, anaerobic bacteria are dominant in the fermentation chambers. The *γ-Proteobacteria* is the major type in anaerobic cultivable bacteria and even the only type in aerobic cultivable bacteria. In future, our research work may be useful to find out the role of bacterial taxa with fermentative characteristics by aiding host with nutrition and digestion.
